# Protein subcellular localization prediction based on compartment-specific features and structure conservation

**DOI:** 10.1186/1471-2105-8-330

**Published:** 2007-09-08

**Authors:** Emily Chia-Yu Su, Hua-Sheng Chiu, Allan Lo, Jenn-Kang Hwang, Ting-Yi Sung, Wen-Lian Hsu

**Affiliations:** 1 Bioinformatics Program, Taiwan International Graduate Program, Academia Sinica, Taipei, Taiwan; 2 Institute of Bioinformatics, National Chiao Tung University, Hsinchu, Taiwan; 3 Bioinformatics Lab., Institute of Information Science, Academia Sinica, Taipei, Taiwan; 4 Department of Life Sciences, National Tsing Hua University, Hsinchu, Taiwan

## Abstract

**Background:**

Protein subcellular localization is crucial for genome annotation, protein function prediction, and drug discovery. Determination of subcellular localization using experimental approaches is time-consuming; thus, computational approaches become highly desirable. Extensive studies of localization prediction have led to the development of several methods including composition-based and homology-based methods. However, their performance might be significantly degraded if homologous sequences are not detected. Moreover, methods that integrate various features could suffer from the problem of low coverage in high-throughput proteomic analyses due to the lack of information to characterize unknown proteins.

**Results:**

We propose a hybrid prediction method for Gram-negative bacteria that combines a one-versus-one support vector machines (SVM) model and a structural homology approach. The SVM model comprises a number of binary classifiers, in which biological features derived from Gram-negative bacteria translocation pathways are incorporated. In the structural homology approach, we employ secondary structure alignment for structural similarity comparison and assign the known localization of the top-ranked protein as the predicted localization of a query protein. The hybrid method achieves overall accuracy of 93.7% and 93.2% using ten-fold cross-validation on the benchmark data sets. In the assessment of the evaluation data sets, our method also attains accurate prediction accuracy of 84.0%, especially when testing on sequences with a low level of homology to the training data. A three-way data split procedure is also incorporated to prevent overestimation of the predictive performance. In addition, we show that the prediction accuracy should be approximately 85% for non-redundant data sets of sequence identity less than 30%.

**Conclusion:**

Our results demonstrate that biological features derived from Gram-negative bacteria translocation pathways yield a significant improvement. The biological features are interpretable and can be applied in advanced analyses and experimental designs. Moreover, the overall accuracy of combining the structural homology approach is further improved, which suggests that structural conservation could be a useful indicator for inferring localization in addition to sequence homology. The proposed method can be used in large-scale analyses of proteomes.

## Background

The prediction of protein subcellular localization (PSL) focuses on determining localization sites of unknown proteins in a cell. The study of PSL is important for elucidating protein functions involved in various cellular processes. Despite recent technical advances, experimental determination of PSL remains time-consuming and labor-intensive. In addition, researches in the post-genomic era have yielded a tremendous amount of sequence data. Given the size and complexity of the data, many researchers would prefer to use prediction systems to identify and screen possible candidates for further analyses. Hence, computational approaches have become increasingly important.

### Previous works

Extensive studies of PSL prediction have led to the development of several methods, which can be classified as follows.

1. *Amino acid composition-based methods *These methods utilize machine learning techniques, including neural networks [1] and support vector machines (SVM) [2-8]. This category includes methods like P-CLASSIFIER [6] and CELLO [7,8], which utilize *n*-peptide composition-based SVM approaches.

2. *Methods that integrate various protein characteristics *Several methods including expert systems [9,10], *k*-nearest neighbor [11-13], SVM [14-16], support vector data description [17], and Bayesian networks [18-21], integrate various biological features that influence localization. The features that characterize a protein can be extracted from biological literature, public databases, and related prediction systems. Both PSORTb [18,19] and PSLpred [14] integrate different analytical modules and demonstrate that the hybrid approaches perform better than each individual module.

3. *Sequence homology-based methods *It has been suggested that PSL is an evolutionary conserved trait [22,23]. Efforts to address the relationship between evolutionary information and localization identity have relied heavily on exploiting sequence similarity to infer PSL. Such methods include phylogenetic profiling [24], domain projection [25], and a sequence homology-based method [7]. Several other methods, such as PSORTb and PSLpred, also incorporate such sequence homology-based components in their analyses.

### Our contributions

The prediction of PSL presents several challenges. First, the performance of amino acid composition-based and sequence homology-based methods might be significantly degraded if homologous sequences are not detected. Second, the results of these two methods are generally difficult to interpret; therefore, it is difficult to determine which biological features should be used to identify specific PSL and why they work well for prediction. If the features were biologically interpretable, the resultant knowledge could help in designing artificial proteins with the desired properties. Meanwhile, methods that integrate various features could suffer from the problem of low coverage in high-throughput proteomic analyses due to the lack of information to characterize unknown proteins. Finally, many PSL methods are implemented on redundant training sets, which might lead to overestimation of the predictive performance. Thus, the performance would be significantly lower if redundant sequences were meticulously removed.

In this study, we propose a hybrid method that combines a one-versus-one (1-v-1) SVM model referred to as PSL101 (Protein Subcellular Localization prediction by 1-On-1 classifiers) and a structural homology approach called PSLsse (Protein Subcellular Localization prediction by secondary structure element alignment) to predict the PSL for Gram-negative bacteria. PSL101 comprises a number of binary classifiers, where compartment-specific biological features derived from Gram-negative bacteria translocation pathways are incorporated. In PSLsse, we employ secondary structure alignment for structural similarity comparison and assign the known localization of the top-ranked protein as the predicted localization of a query protein. Experiment results show that PSL101 achieves high prediction accuracy, which demonstrates that biological features derived from Gram-negative bacteria translocation pathways significantly enhance the performance. Moreover, since the selected features are biologically interpretable, they can be easily applied to advanced analyses and experimental designs. Most notably, the overall accuracy of combining PSL101 and PSLsse is further improved to 93.7%, which is a 2.5% improvement over the second best method. Our analysis suggests that, in addition to sequence homology, structural homology can also be an effective indicator for inferring PSL. Lastly, since sequence redundancy in the training data often leads to overestimation of prediction accuracy, we present an evaluation using non-redundant data sets. It is also known that cross-validation may overestimate the predictive performance when parameters are optimized repeatedly on the same test data. Therefore, we adopt a three-way data split procedure for evaluating the non-redundant data sets. The results suggest that these techniques can prevent overestimation of the performance such that the general performance of PSL prediction should be approximately 85%. In the assessment of the evaluation data sets, our hybrid method also provides accurate prediction, especially for those sequences of low homology to the training set.

## Results and discussion

### Data sets

To assess our method, we utilize several data sets of Gram-negative bacteria proteins that have been used in previous works [6-8,14,18,19]. Gram-negative bacteria have five major PSL sites: the cytoplasm (CP), inner membrane (IM), periplasm (PP), outer membrane (OM), and extracellular space (EC). Table 1 lists the number of proteins in different localization sites in the data sets, which are detailed in Table 1S of the supplementary material [see Additional file 1].

**Table 1:** Number of proteins distributed in different localization sites in the data sets.

Localization	Benchmark		Non-redundant		Evaluation		
			
	PS1302	PS1444	NR755	NR828	EV90_high	EV153_low	EV243_all
Cytoplasm (CP)	248	278	206	229	28	96	124
Inner membrane (IM)	268	309	182	205	26	26	52
Periplasm (PP)	244	276	147	161	13	11	24
Outer membrane (OM)	352	391	134	148	19	9	28
Extracellular space (EC)	190	190	86	85	4	11	15

Total	1302	1444	755	828	90	153	243

1. Benchmark data sets: Derived from the first release of ePSORTdb [26], the first data set, referred to as PS1302, consists of proteins with experimentally determined localizations. The second data set, PS1444, is an expanded version of PS1302.

2. Non-redundant data sets: To assess the predictive performance of non-homologous proteins, we utilize CD-HIT [27], a redundancy filtering program, to eliminate sequences that share greater or equal to 30% sequence identity in the PS1302 and PS1444 data sets, which yields the NR755 and NR828 data sets, respectively.

3. Evaluation data sets: Recently, a new data set [22] comprised of 299 proteins was created for comparison of different methods. We first apply ClustalW [28] to divide the new set into two subsets according to the sequence identity of each protein pair between the 299 proteins and proteins in the known training sets (i.e., PS1302 and PS1444) with a cutoff of 30%. Then, redundant sequences are removed from each subset by CD-HIT with a 30% threshold; the resultant non-redundant data sets are called EV90_high (≧30%) and EV153_low (<30%). The combination of both sets is referred to as the EV243_all data set.

### Effect of biological features derived from Gram-negative bacteria translocation pathways

Since it is impractical to try all possible feature combinations in different classifiers, heuristics guided by biological insights are used to determine a small subset of feature sets specific to each classifier. Starting with an empty subset, a sequential forward search algorithm [29] keeps adding the best feature sets that improve the accuracy. The process terminates when adding a feature set no longer makes any improvement. The performance of PSL101 evaluated by ten-fold cross-validation for the benchmark data sets is shown in the leftmost column of Table 2. PSL101 attains overall accuracy of 92.7% and 91.6% for the PS1302 and PS1444 data sets, respectively. Most notably, CP and IM proteins attain accurate prediction performance in terms of both accuracy and *MCC*, which can be explained by the fact that proteins localized in CP and IM are characterized by several well-known biological features in our method.

**Table 2:** Comparison of different hybrid approaches using cross-validation for the benchmark data sets.

	PS1302					
	
Localization	PSL101		PSLseq+PSL101		PSLsse+PSL101	
			
	*Acc *(%)	*MCC*	*Acc *(%)	*MCC*	*Acc *(%)	*MCC*
CP	97.2 (94.8)	0.91 (0.89)	96.4 (94.4)	0.90 (0.89)	95.6 (94.4)	0.90 (0.90)
IM	94.4 (92.9)	0.95 (0.94)	93.3 (91.8)	0.95 (0.93)	93.3 (91.8)	0.94 (0.93)
PP	87.7 (88.1)	0.86 (0.84)	88.9 (88.9)	0.86 (0.85)	91.4 (91.0)	0.88 (0.88)
OM	94.3 (93.8)	0.94 (0.91)	95.5 (95.7)	0.96 (0.93)	96.3 (96.9)	0.96 (0.95)
EC	87.9 (83.2)	0.87 (0.84)	89.5 (85.8)	0.89 (0.87)	90.0 (87.9)	0.89 (0.89)

Overall	92.7 (91.2)	-	93.1 (91.9)	-	93.7 (92.9)	-

	PS1444					
	
Localization	PSL101		PSLseq+PSL101		PSLsse+PSL101	
			
	*Acc *(%)	*MCC*	*Acc *(%)	*MCC*	*Acc *(%)	*MCC*

CP	96.0 (94.2)	0.91 (0.90)	94.6 (92.8)	0.89 (0.88)	95.0 (93.5)	0.91 (0.90)
IM	94.5 (92.6)	0.95 (0.94)	93.5 (91.6)	0.94 (0.93)	93.5 (91.6)	0.94 (0.93)
PP	85.1 (88.0)	0.82 (0.83)	87.0 (88.4)	0.84 (0.83)	90.2 (91.7)	0.86 (0.87)
OM	94.9 (93.9)	0.93 (0.91)	95.9 (95.7)	0.95 (0.93)	96.7 (96.4)	0.96 (0.95)
EC	82.6 (83.2)	0.83 (0.85)	87.9 (86.3)	0.87 (0.88)	87.4 (87.9)	0.87 (0.89)

Overall	91.6 (91.1)	-	92.4 (91.6)	-	93.2 (92.8)	-

§ The performance of incorporating a three-way data split procedure is indicated in the parentheses.

**Figure 1:**
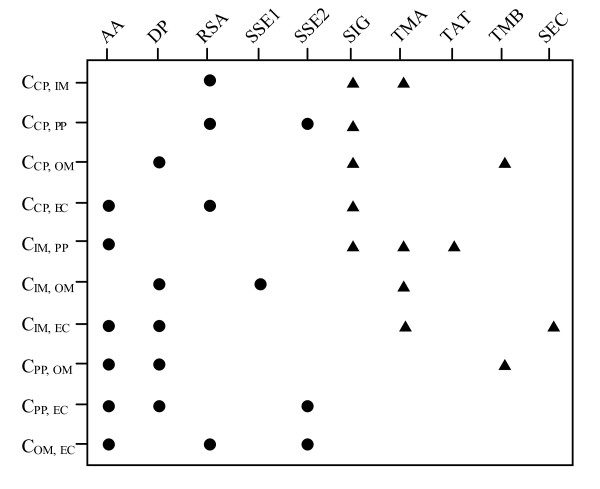
**Feature combinations derived from the PS1302 data set using cross-validation**. Selected general and compartment-specific features are represented by filled circles and triangles, respectively.

The features selected from PSL101 for the PS1302 data set using cross-validation are shown in Figure 1; the same set of features is used in the corresponding training and testing scheme for the PS1444 data set. The experiment results demonstrate that our feature selection not only yields a significant improvement in the performance, but also correlates well with biological insights. For example, in Figure 1, PSL101 selects signal peptides, transmembrane *a*-helices, and relevant solvent accessibility (i.e. SIG, TMA, and RSA) as the optimal features to distinguish CP and IM proteins. In addition, di-peptide composition, signal peptides, and transmembrane *β*-barrels (i.e. DP, SIG, and TMB) are used in the discrimination of CP and OM proteins. The combination of general and compartment-specific features works well in differentiating between any two compartments in each classifier; accordingly, the overall accuracy of the combined predictions of each classifier is improved. The results support our assumption that compartment-specific biological features derived from Gram-negative bacteria translocation pathways can significantly enhance the performance of PSL prediction. Moreover, the selected features are biologically interpretable and can be easily applied in further analyses.

### Effect of sequence and structure conservation

**Figure 2:**
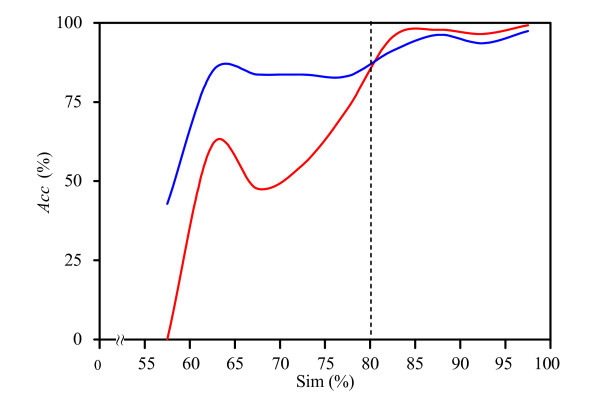
**The distribution of the prediction accuracy as a function of secondary structure similarity**. The blue line and the red line indicate the distribution of the prediction accuracy as a function of secondary structure similarity for PSL101 and PSLsse using cross-validation for the PS1444 data set, respectively.

We now explore the relationship between sequence and structural similarity and localization identity. Both sequence and structural homology approaches, referred to as PSLseq and PSLsse, are developed to infer localization based on sequence alignment using ClustalW and secondary structure alignment using SSEA [30], respectively. Figure 2 shows that when the structural similarity is greater or equal to 80%, PSLsse performs slightly better than PSL101; otherwise, PSL101 is significantly better. Thus, we propose a hybrid approach that combines PSLsse and PSL101, called PSLsse+PSL101. For each query protein, if the top-rank aligned protein shares an 80% or greater structural similarity with any of the proteins in the training set, the localization is predicted by PSLsse; otherwise, it is predicted by PSL101. In addition, we implement another hybrid approach, called PSLseq+PSL101, which uses a cutoff of 30% sequence identity [7] to combine PSLseq and PSL101.

Table 2 compares the performance of different hybrid approaches using ten-fold cross-validation for the benchmark data sets. Compared with PSL101, the performance of the two hybrid approaches, PSLseq+PSL101 and PSLsse+PSL101, is significantly enhanced in terms of the overall accuracy, as well as the accuracy and *MCC *of most localization sites. Most notably, the accuracy of EC proteins in both data sets is improved by 1.6%~5.3%, which suggests that homology-based approaches can compensate for the performance of PSL101 and thereby enhance the prediction of EC proteins. Moreover, PSLsse+PSL101 achieves an overall accuracy of 93.7% and 93.2% in the PS1302 and PS1444 data sets, respectively, which are 0.6%~0.8% improvements over PSLseq+PSL101. We show that homology approaches based on sequence and structure conservation work well in PSL prediction; in fact, structural homology could be effective for prediction in addition to sequence homology. Thus, it could also be a useful indicator for inferring PSL.

### Performance comparison of *n*-fold cross validation and three-way data split

The performance of the three-way data split experiments is shown in parentheses in Table 2. The features selected from PSL101 for the PS1302 data set using three-way data split are shown in Figure 1S in the supplementary material [see Additional file 1]; the same set of features is used in the corresponding training and testing scheme for the PS1444 data set. The overall accuracy of PSL101, PSLseq+PSL101, and PSLsse+PSL101 drop 0.4%~1.5% for both the PS1302 and PS1444 data sets. Specifically, the accuracy and *MCC *of the same localization sites are consistent across the two different data sets. Moreover, the performance of the two data sets evaluated using three-way data split is more consistent than that assessed by ten-fold cross-validation. This suggests that a three-way data split procedure could avoid overestimation of the predictive performance; therefore, it should be considered in PSL prediction.

### Comparison with other approaches using the benchmark data sets

Table 3 compares the performance of PSLsse+PSL101, referred to as HYBRID, with other prediction methods using cross-validation on the benchmark data sets. HYBRID attains the best overall accuracy of 93.7% and 93.2% for the PS1302 and PS1444 data sets, respectively. In both sets, HYBRID achieves improvements of 2.5%~3.2% in overall accuracy compared to the second best approaches in each data set. With respect to accuracy and *MCC*, HYBRID performs better than the other approaches in most localization sites. HYBRID ranks the best in terms of accuracy for CP, IM, and OM proteins, in which more biological features are incorporated than the other localization sites. The high predictive performance for CP, IM, and OM proteins demonstrates that biological features derived from Gram-negative bacteria translocation pathways are effective for PSL prediction. Most notably, it outperforms the second best approaches for IM proteins by 4.9~14.6% and 0.9~3.5% in terms of accuracy for the PS1302 and PS1444 data sets, respectively. This is a particular strength of HYBRID because IM proteins constitute the key components of various cellular processes and serve as important targets for drug discovery [31]. In addition, it is interesting to note that the accuracy of IM proteins is significantly improved from 78.7% in PSORTb v.1.1 to 92.6% in PSORTb v.2.0, in which an expanded homology module is incorporated. This also lends support on our assumption that sequence and structural homology approaches could be effective indicators for inferring PSL.

**Table 3:** Performance comparison of different approaches using cross-validation for the benchmark data sets.

	PS1302									
	
Localization	HYBRID		CELLO		PSORTb v.1.1		PSLpred		P-CLASSIFIER	
					
	*Acc *(%)	*MCC*	*Acc *(%)	*MCC*	*Acc *(%)	*MCC*	*Acc *(%)	*MCC*	*Acc *(%)	*MCC*
CP	95.6	0.90	90.7	0.85	69.4	0.79	90.7	0.86	94.6	0.85
IM	93.3	0.94	88.4	0.92	78.7	0.85	86.8	0.88	87.1	0.92
PP	91.4	0.88	86.9	0.80	57.6	0.69	90.3	0.90	85.9	0.81
OM	96.3	0.96	94.6	0.90	90.3	0.93	95.2	0.95	93.6	0.90
EC	90.0	0.89	78.9	0.82	70.0	0.79	90.6	0.84	86.0	0.89

Overall	93.7	-	88.9	-	74.8	-	91.2	-	89.8	-

	PS1444									
	
Localization	HYBRID		CELLO II		PSORTb v.2.0		PSLpred		P-CLASSIFIER	
					
	*Acc *(%)	*MCC*	*Acc *(%)	*MCC*	*Acc *(%)	*MCC*	*Acc *(%)	*MCC*	*Acc *(%)	*MCC*

CP	95.0	0.91	95.3	0.89	70.1	0.77	-	-	-	-
IM	93.5	0.94	90.0	0.91	92.6	0.92	-	-	-	-
PP	90.2	0.86	87.7	0.82	69.2	0.78	-	-	-	-
OM	96.7	0.96	92.8	0.90	94.9	0.95	-	-	-	-
EC	87.4	0.87	79.5	0.82	78.9	0.86	-	-	-	-

Overall	93.2	-	90.0	-	82.6	-	-	-	-	-

§ The best performance of overall and individual localization sites is underlined.

### Comparison with other approaches using the evaluation data sets

The evaluation data sets were submitted to the web servers of each prediction method. The predictive performance is shown in Table 4. CELLO II and P-CLASSIFIER achieve consistent overall accuracy in the range of 71.9%~77.8% for the EV90_high and EV153_low data sets. PSLpred attains overall accuracy of 72.5% and 88.9% for the EV153_low and EV90_high sets, respectively. PSORTb v.2.0 performs very well for the EV90_high set, but poorly for the EV153_low set. HYBRID yields the best predictions for proteins of low sequence similarity and ranks second best for highly homologous sequences. This demonstrates that when no homologous sequences are detected, biological features derived from Gram-negative bacteria translocation pathways yield accurate prediction; on the other hand, the incorporation of structural homology approach further improves the predictive performance for highly homologous sequences. When both data sets are evaluated on the EV243_all set, HYBRID achieves an overall accuracy of 84.0%, which is a 5.4% improvement over the second best method. This suggests that HYBRID could enhance the robustness of PSL prediction, especially when highly homologous sequences are not detected.

**Table 4:** Predictive performance of different prediction methods for the evaluation data sets.

	EV153_low									
	
Localization	HYBRID		CELLO II		PSORTb v.2.0		PSLpred		P-CLASSIFIER	
					
	*Acc *(%)	*MCC*	*Acc *(%)	*MCC*	*Acc *(%)	*MCC*	*Acc *(%)	*MCC*	*Acc *(%)	*MCC*
CP	91.7	0.67	91.7	0.70	63.5	-0.61	89.6	0.59	91.7	0.66
IM	65.4	0.73	46.2	0.64	46.2	-0.58	38.5	0.41	30.8	0.48
PP	45.5	0.25	81.8	0.49	00.0	-0.03	54.5	0.34	81.8	0.49
OM	44.4	0.58	33.3	0.34	22.2	-0.46	44.4	0.58	22.2	0.17
EC	27.3	0.43	45.5	0.50	09.1	-0.29	45.5	0.54	27.3	0.33

Overall	76.5	-	76.5	-	49.7	-	72.5	-	71.9	-

	EV90_high									
	
Localization	HYBRID		CELLO II		PSORTb v.2.0		PSLpred		P-CLASSIFIER	
					
	*Acc *(%)	*MCC*	*Acc *(%)	*MCC*	*Acc *(%)	*MCC*	*Acc *(%)	*MCC*	*Acc *(%)	*MCC*

CP	100.0	0.95	92.9	0.83	100.0	1.00	096.4	0.88	92.9	0.78
IM	96.2	0.97	73.1	0.75	100.0	1.00	92.3	0.92	80.8	0.84
PP	100.0	0.96	61.5	0.58	100.0	1.00	92.3	0.83	46.2	0.46
OM	94.7	0.97	73.7	0.67	94.7	0.97	68.4	0.79	73.7	0.69
EC	75.0	0.86	75.0	0.54	100.0	1.00	100.0	0.81	75.0	0.54

Overall	96.7	-	77.8	-	98.9	-	88.9	-	77.8	-

	EV243_all									
	
Localization	HYBRID		CELLO II		PSORTb v.2.0		PSLpred		P-CLASSIFIER	
					
	*Acc *(%)	*MCC*	*Acc *(%)	*MCC*	*Acc *(%)	*MCC*	*Acc *(%)	*MCC*	*Acc *(%)	*MCC*

CP	93.5	0.80	91.9	0.77	71.8	0.73	91.1	0.72	91.9	0.73
IM	80.8	0.85	59.6	0.70	73.1	0.80	65.4	0.68	55.8	0.67
PP	75.0	0.56	70.8	0.51	54.2	0.66	75.0	0.57	62.5	0.45
OM	78.6	0.85	60.7	0.58	71.4	0.83	60.7	0.73	57.1	0.53
EC	40.0	0.57	53.3	0.50	33.3	0.57	60.0	0.62	40.0	0.39

Overall	84.0	-	77.0	-	67.9	-	78.6	-	74.1	-

§ The best performance of overall and individual localization sites is underlined. HYBRID is trained on the PS1444 data set.

### Performance of non-redundant data sets

In both benchmark data sets, proteins sharing up to 30% sequence identity comprise approximately 42% of the sets. One drawback of a high level of redundancy in data sets is that it could lead to poor generalization for a predictor, since the predictor might fail to assign a correct PSL, especially for those sequences of low homology to the training set. For this reason, the construction of non-redundant data sets is necessary when evaluating the performance of PSL prediction.

Here, we present performance assessments using non-redundant sequences from Gram-negative bacteria data sets. Using the same features derived from the PS1302 set by cross-validation, we use HYBRID to train and evaluate the two non-redundant sets via ten-fold cross-validation. The performance is shown in Table 5. The overall accuracy declines markedly by approximately 8% using the non-redundant sets compared with those using the redundant sets. The *MCC *for individual localization sites also drops by 0.04~0.26. These results indicate that the general performance of PSL prediction for Gram-negative bacteria is approximately 85% for non-redundant data sets. Methods that are less dependent on homology detection should be developed if highly homologous sequences are removed completely.

**Table 5:** Performance of non-redundant data sets.

Localization	NR755		NR828	
	
	*Acc *(%)	*MCC*	*Acc *(%)	*MCC*
CP	95.6	0.86	97.8	0.87
IM	88.5	0.88	88.8	0.90
PP	81.0	0.76	80.7	0.76
OM	85.1	0.84	83.8	0.82
EC	64.0	0.65	57.6	0.61

Overall	85.6	-	85.6	-

## Conclusion

In this paper, we have proposed a hybrid method for predicting PSL for Gram-negative bacteria based on a combination of a 1-v-1 SVM model using compartment-specific biological features and a structural homology approach using secondary structure alignment. Experiment results show that the SVM model achieves high prediction accuracy for both benchmark data sets, thus supporting the assumption that biological features derived from Gram-negative bacteria translocation pathways could significantly improve the performance. The overall accuracy of combining the SVM model and the structural homology approach is further improved, which indicates that structural homology, like sequence homology, could also be a useful indicator for inferring PSL. A three-way data split procedure is incorporated to prevent overfitting of the parameters and features. In addition, non-redundant data sets have been used for the evaluation of Gram-negative bacteria. The results suggest that the performance could be overestimated if redundant sequences are considered. In the assessment of the evaluation data sets, our hybrid method provides accurate predictions, especially when sequences of low sequence similarity to the training data are detected. The proposed method can be used in large-scale analyses of proteomes and is freely available for public use at [32].

There are still some challenges to be addressed in PSL prediction. In our work, we only consider proteins with single localization sites. However, proteins with multiple localization sites are not a rarity, especially in higher order species [33,34]. In our future development, we will consider those proteins localized to multiple compartments. In addition, better accuracy and coverage are needed, particularly for several poorly predicted localization sites. We will also extend our method to combine more biological features, analyze multiple compartment proteins, and incorporate proteins of more species, including those of humans.

## Methods

### Gram-negative bacteria translocation pathways

**Figure 3:**
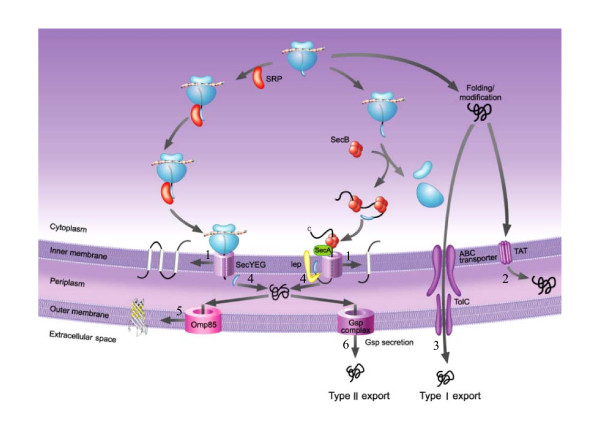
**Diversity of Gram-negative bacteria translocation pathways**. 1, 2, 3, 4, 5, and 6 represent translocation pathways from CP to IM, CP to PP, CP to EC, IM to PP, PP to OM, and PP to EC, respectively. SRP, signal recognition particle, SecB, export-specific cytoplasmic chaperone, SecA, preprotein translocase SecA subunit, SecYEG, preprotein translocase complex, lep, leader peptidase, TAT, twin argine translocase, Gsp complex, general secretion pathway complex, Omp85, outer membrane protein assembly factor, ABC transporter, ATP-binding cassette transporter, TolC, Type I secretion outer membrane protein. [Modified from Wickner and Schekman (2005) with permission]

Proteins synthesized in the cytosol must be targeted and transported to their designated compartments in Gram-negative bacteria through one of the translocation pathways [35]. Gram-negative bacteria have five major PSL sites, which are the CP, IM, PP, OM, and EC. Figure 3 shows some of the translocation pathways in Gram-negative bacteria. Translocations through the IM are targeted, both co-translationally and post-translationally, to the SecYEG translocase via the signal recognition particle (SRP)-dependent pathways and the SecB-dependent pathways, respectively. Alternatively, proteins localized to the PP can cross the IM by the twin arginine translocation pathway. PP proteins can be inserted or translocated across the OM through five secretory pathways, including Type I [36] and Type II [37] export systems. Regardless of the mode of translocation, the process is largely substrate specific, and therefore requires one or more signals in order to cross a membrane. For example, non-cytoplasmic proteins contain signal sequences that direct them to translocate through the IM. Furthermore, many proteins localized to a compartment have characteristic structures and amino acid compositions. Integral IM proteins contain mainly transmembrane *a*-helices, in which their cores are populated by hydrophobic residues. Therefore, we model the prediction system according to the translocation pathways by identifying signals that influence the targeting and compartment-specific features that correlate with various localization sites.

### System architecture of PSL101

**Figure 4:**
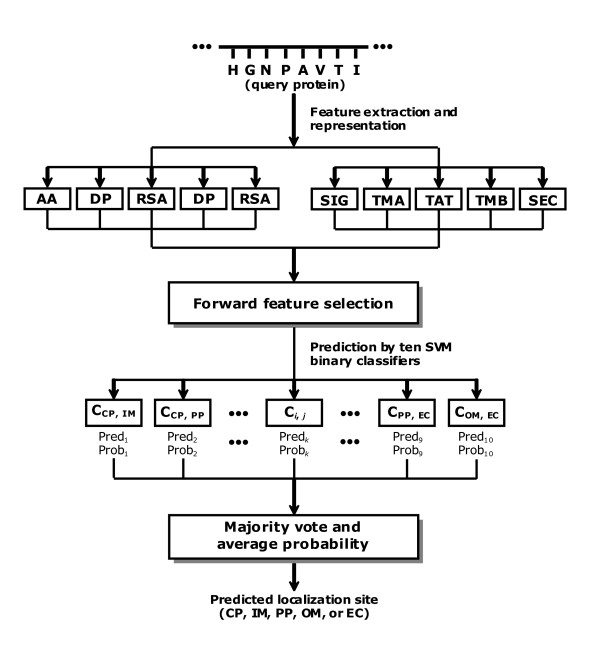
System architecture of PSL101.

The system architecture of PSL101, shown in Figure 4, comprises ten binary 1-v-1 SVM [38] classifiers for the prediction of five localization sites of Gram-negative bacteria. Each translocation step across compartments *i *and *j *is represented by a binary classifier *C*_*i*,*j *_in which different biological features intrinsic to the proteins in compartments *i *and *j *are incorporated. All translocations in Figure 3, i.e., translocation pathways 1 to 6, can be modelled in this way by using six binary classifiers. The remaining four classifiers, although not biologically occurring, are still constructed with compartment-specific features and combined with the above classifiers for an integrated prediction. For each query protein, a predicted class and its corresponding probability are returned by each classifier. To determine the predicted localization site of the protein, we combine the results of the ten binary classifiers based on majority vote. In the case of a tie, the localization site with the highest average probability is assigned as the final prediction result.

### Feature extraction and representation of PSL101

We consider the following biological features to distinguish between proteins translocated to different compartments, and construct our classification framework to mimic the translocation process of Gram-negative bacterial secretory pathways. Since some of these features may not be readily available, we utilize several web services to predict them.

#### General biological features

1. Amino acid (AA) composition: Protein descriptors based on *n*-peptide compositions or their variations have proved effective in PSL prediction [8]. If *n *= 1, then the *n*-peptide composition reduces to amino acid composition, which generates a 21 dimensional feature vector (i.e., 20 amino acid types plus a symbol 'X', for others) that represents the occurrence frequency of amino acids in a protein sequence.

2. Di-peptide (DP) composition: Similar to amino acid composition, if *n *= 2, the di-peptide composition gives a fixed length of 21 × 21 di-peptides, which represent the occurrence frequency of amino acid pairs in a protein sequence.

3. Relative solvent accessibility (RSA): Proteins in different compartments have various buried and exposed residue compositions [39,40]. For example, CP proteins have a balance of acidic and basic surface residues, while EC proteins have a slight excess of acidic surface residues [41]. We use amino acid compositions of both buried and exposed residues, with a cutoff of 25% [42], to represent the results derived by SABLE II [43], a relative solvent accessibility prediction method.

4. Secondary structure elements encoding scheme 1 (SSE1): Transmembrane *a*-helices are frequently observed in IM proteins, while transmembrane *β*-barrels are primarily found in OM proteins [44]. Secondary structure elements (SSE) are crucial for detecting proteins localized in the IM and OM. We compute the amino acid compositions of three SSEs [15,40], *a*-helix, *β*-strand, and loop, based on the predictions of HYPROSP II [45], a knowledge-based SSE prediction approach.

5. Secondary structure elements encoding scheme 2 (SSE2): SSE1 alone cannot discriminate proteins that share similar SSE compositions and localize in different compartments. For example, the SSE compositions of OM proteins might be similar to proteins localized in other compartments, but OM proteins are characterized by *β*-strand repeats throughout the transmembrane domains. To further depict such properties in a protein, three descriptors, composition, transition, and distribution, are used to encode predictions of HYPROSP II. Composition describes the global composition of a given SSE type in a protein. Transition characterizes the percentage frequency that amino acids of a particular SSE type are followed by a different type. Distribution measures the chain length within which the first, 25, 50, 75 and 100% of the amino acids of a particular SSE type are located [46]. An example is shown in Figure 2S in the supplementary material [see Additional file 1].

#### Compartment-specific biological features

1. Signal peptides (SIG): Signal peptides are N-terminal peptides, typically between 15 and 40 amino acids long, which target proteins for translocation through the general secretory pathway [1]. The presence of a signal peptide suggests that the protein does not reside in the CP and several prediction methods have been developed [47-49]. We employ SignalP 3.0 [47], a neural network- and hidden Markov model-based method, to predict the presence and location of signal peptide cleavage sites.

2. Transmembrane *a*-helices (TMA): Integral IM proteins are characterized by *a*-helices, typically 20–25 amino acids in length, which traverse the IM. The presence of one or more transmembrane *a*-helices implies that the protein is located in the IM. We apply TMHMM 2.0 [50], a hidden Markov model-based method, to identify potential transmembrane *a*-helices.

3. Twin-arginine translocase (TAT) motifs: The twin-arginine translocase system exports proteins from the CP to the PP. The proteins translocated by twin-arginine translocase bear a unique twin-arginine motif [51], the presence of which is a useful feature for distinguishing between PP and non-PP proteins. We use TatP 1.0 [52], a neural network-based method, to predict the presence of twin-arginine translocase motifs.

4. Transmembrane *β*-barrels (TMB): A large number of proteins residing in the OM are characterized by *β*-barrel structures; thus, they could be candidate features for detecting OM proteins. We adopt TMB-Hunt [53], a method that uses a *k*-nearest neighbor algorithm, to distinguish between transmembrane *β*-barrels and non-transmembrane *β*-barrels.

5. Non-classical protein secretion (SEC): For a long time, it was believed that an N-terminal signal peptide was absolutely necessary to export a protein to the extracellular space. However, recent studies have shown that several EC proteins can be secreted without a classical N-terminal signal peptide [54]. Identification of non-classical protein secretion could be a potential discriminator for CP and EC proteins. Predictions from SecretomeP 2.0 [55], a non-classical protein secretion prediction method, are incorporated in our method.

### Sequence and structure conservation

Because PSL tends to be evolutionary conserved, the known localization sites of homologous sequences could be useful indicators of the actual localization of an unknown protein. We apply both sequence and structural homology approaches to infer localization. For the sequence homology approach, we develop a prediction method, called PSLseq, which is based on pairwise sequence alignment of ClustalW. In the structural homology approach, we employ secondary structural similarity comparison, referred to as PSLsse. Based on secondary structure elements predicted by HYPROSP II, we use SSEA to perform pairwise secondary structure alignment. In the sequence and structural homology approaches, the known localization of the top-rank aligned protein is assigned to the query protein as its predicted localization.

### Performance assessment

For comparison with other approaches, we follow the measures used in previous works [6-8,14]. To assess the performance in each localization class, the accuracy and Matthew's correlation coefficient (*MCC*) [56] are calculated by Equations (1) and (2), respectively. The overall accuracy is defined in Equation (3).

*Acc*_*i *_= (*TP*_*i*_/*N*_*i*_) × 100%

MCCi=TPi×TNi−FPi×FNi(TPi+FNi)(TPi+FPi)(TNi+FPi)(TNi+FNi)
 MathType@MTEF@5@5@+=feaafiart1ev1aaatCvAUfKttLearuWrP9MDH5MBPbIqV92AaeXatLxBI9gBaebbnrfifHhDYfgasaacH8akY=wiFfYdH8Gipec8Eeeu0xXdbba9frFj0=OqFfea0dXdd9vqai=hGuQ8kuc9pgc9s8qqaq=dirpe0xb9q8qiLsFr0=vr0=vr0dc8meaabaqaciaacaGaaeqabaqabeGadaaakeaacqWGnbqtcqWGdbWqcqWGdbWqdaWgaaWcbaGaemyAaKgabeaakiabg2da9maalaaabaGaemivaqLaemiuaa1aaSbaaSqaaiabdMgaPbqabaGccqGHxdaTcqWGubavcqWGobGtdaWgaaWcbaGaemyAaKgabeaakiabgkHiTiabdAeagjabdcfaqnaaBaaaleaacqWGPbqAaeqaaOGaey41aqRaemOrayKaemOta40aaSbaaSqaaiabdMgaPbqabaaakeaadaGcaaqaamaabmaabaGaemivaqLaemiuaa1aaSbaaSqaaiabdMgaPbqabaGccqGHRaWkcqWGgbGrcqWGobGtdaWgaaWcbaGaemyAaKgabeaaaOGaayjkaiaawMcaamaabmaabaGaemivaqLaemiuaa1aaSbaaSqaaiabdMgaPbqabaGccqGHRaWkcqWGgbGrcqWGqbaudaWgaaWcbaGaemyAaKgabeaaaOGaayjkaiaawMcaamaabmaabaGaemivaqLaemOta40aaSbaaSqaaiabdMgaPbqabaGccqGHRaWkcqWGgbGrcqWGqbaudaWgaaWcbaGaemyAaKgabeaaaOGaayjkaiaawMcaamaabmaabaGaemivaqLaemOta40aaSbaaSqaaiabdMgaPbqabaGccqGHRaWkcqWGgbGrcqWGobGtdaWgaaWcbaGaemyAaKgabeaaaOGaayjkaiaawMcaaaWcbeaaaaaaaa@6FBA@

Acc=(∑i=1lTPi/∑i=1lNi)×100%
 MathType@MTEF@5@5@+=feaafiart1ev1aaatCvAUfKttLearuWrP9MDH5MBPbIqV92AaeXatLxBI9gBaebbnrfifHhDYfgasaacH8akY=wiFfYdH8Gipec8Eeeu0xXdbba9frFj0=OqFfea0dXdd9vqai=hGuQ8kuc9pgc9s8qqaq=dirpe0xb9q8qiLsFr0=vr0=vr0dc8meaabaqaciaacaGaaeqabaqabeGadaaakeaacqWGbbqqcqWGJbWycqWGJbWycqGH9aqpdaqadaqaamaalyaabaWaaabCaeaacqWGubavcqWGqbaudaWgaaWcbaGaemyAaKgabeaaaeaacqWGPbqAcqGH9aqpcqaIXaqmaeaacqWGSbaBa0GaeyyeIuoaaOqaamaaqahabaGaemOta40aaSbaaSqaaiabdMgaPbqabaaabaGaemyAaKMaeyypa0JaeGymaedabaGaemiBaWganiabggHiLdaaaaGccaGLOaGaayzkaaGaey41aqRaeGymaeJaeGimaaJaeGimaaJaeiyjaucaaa@4D22@

where *l *= 5 is the total number of localization sites for Gram-negative bacteria, and *TP*_*i*_, *TN*_*i*_, *FP*_*i*_, *FN*_*i*_, and *N*_*i *_are, respectively, the number of true positives, true negatives, false positives, false negatives, and proteins in localization site *i*. *MCC*, which considers both under- and over-predictions, provides a complementary measure of the predictive performance, where *MCC *= 1 indicates a perfect prediction, *MCC *= 0 indicates a completely random assignment, and *MCC *= -1 indicates a perfectly reverse correlation.

### Training and testing

We apply the LIBSVM [57] software in our experiments. For all classifiers, we use the Radial Basis Function kernel, and tune the cost (*c*) and gamma (*γ*) parameters. The probability estimates in LIBSVM are used to determine the confidence levels of the classifications [58]. The performance of PSL101 is assessed as follows.

1. *n*-fold cross-validation: The data set is randomly partitioned into ten distinct non-overlapping sets of proteins (i.e., *n *= 10), nine of which are used to train the predictor. Then, the accuracy of the predictor is evaluated on the remaining set.

2. Three-way data split: To prevent data overfitting, a three-way data split procedure [59] is used to assess the performance of PSL101. The data set is randomly divided into three disjoint sets, i.e., a training set for classifier learning, a validation set for feature selection and parameter tuning, and a test set for performance evaluation. Here, we divide the data set into ten distinct sets: eight for training, one for validation, and one for testing.

It is worth mentioning that among the independent data set tests, *n*-fold cross-validation and jackknife tests are often used for examining the accuracy of statistical prediction methods. The jackknife test is deemed the most rigorous and objective and it has been increasingly adopted by investigators to test the power of various prediction methods as analyzed by a comprehensive review [60]. In our work, we limit ourselves to using *n*-fold cross-validation for suitable comparisons with other methods that also use the same evaluation schemes.

## Authors' contributions

ECYS developed the method, carried out the computational predictions, and drafted the manuscript. HSC and AL participated in the experimental design and refined the manuscript. HSC wrote evaluation programs and performed homology search. AL provided biological knowledge and supplied additional insights regarding the analyses. JKH, TYS, and WLH coordinated the study. All authors read and approved the final manuscript.

## Supplementary Material

Additional file 1 Protein subcellular localization prediction based on compartment-specific features and structure conservation (Supplementary Data). The supplementary material of this study. Click here for file
