# Supplementary material for: Protein subcellular localization prediction based on compartment-specific features and structure conservation
Source: BMC Bioinformatics. 2007 Sep 8;8:330. doi: 10.1186/1471-2105-8-330 (PMC2040162; doi:10.1186/1471-2105-8-330)
Supplement: Additional file 1 — Protein subcellular localization prediction based on compartment-specific features and structure conservation (Supplementary Data). The supplementary material of this study. [file 1471-2105-8-330-S1.pdf]

Protein subcellular localization prediction based on compartment-specific features and structure conservation (Supplementary Data)

Emily Chia-Yu Su[1, 2], Hua-Sheng Chiu[3], Allan Lo[1, 4], Jenn-Kang Hwang[2], Ting-Yi Sung[3], and Wen-Lian Hsu[3, *]

[1] Bioinformatics Program, Taiwan International Graduate Program, Academia Sinica, Taipei, Taiwan

[2] Institute of Bioinformatics, National Chiao Tung University, Hsinchu, Taiwan

[3] Bioinformatics Lab., Institute of Information Science, Academia Sinica, Taipei, Taiwan

[4] Department of Life Sciences, National Tsing Hua University, Hsinchu, Taiwan

Emails: {cysu, huasheng, allanlo}@iis.sinica.edu.tw, jkhwang@cc.nctu.edu.tw, {tsung, hsu}@iis.sinica.edu.tw

* To whom correspondence should be addressed. Tel: +886-2-27883799 ext. 1804; Fax: +886-2-27824814; Email: hsu@iis.sinica.edu.tw

1. DATA SETS

We provide the data sets for protein subcellular localization prediction used in the training and testing of the proposed method. Table 1S lists the links to download the benchmark data sets, the non-redundant data set, and the evaluation data sets described in the main paper.

Table 1S: The benchmark, non-redundant, and evaluation data sets used in PSL101.

Data Set	Number of Proteins	Link
PS1302	1302	http://bio-cluster.iis.sinica.edu.tw/~bioapp/PSL101/dataset/PS1302.fasta
PS1444	1444	http://bio-cluster.iis.sinica.edu.tw/~bioapp/PSL101/dataset/PS1444.fasta
NR755	755	http://bio-cluster.iis.sinica.edu.tw/~bioapp/PSL101/dataset/NR755.fasta
NR828	828	http://bio-cluster.iis.sinica.edu.tw/~bioapp/PSL101/dataset/NR828.fasta
EV90_high	90	http://bio-cluster.iis.sinica.edu.tw/~bioapp/PSL101/dataset/EV90_high.fasta
EV153_low	153	http://bio-cluster.iis.sinica.edu.tw/~bioapp/PSL101/dataset/EV153_low.fasta
EV243_all	243	http://bio-cluster.iis.sinica.edu.tw/~bioapp/PSL101/dataset/EV243_all.fasta

2. SELECTED FEATURE COMBINATIONS

The features selected from PSL101 for the PS1302 data set using a three-way data split procedure are shown in Figure 1S. The selected features correspond well to those discussed in the main paper. In Figure 1S, PSL101 also selects SIG, TMA, and RSA as the optimal features to distinguish cytoplasmic and inner membrane proteins. In addition, RSA, TMA, and SEC are used in the discrimination of proteins localized in the inner membrane and extracellular space. The results indicate that the selected features are highly correlated with the biological insights derived from Gram-negative bacteria translocation pathways.

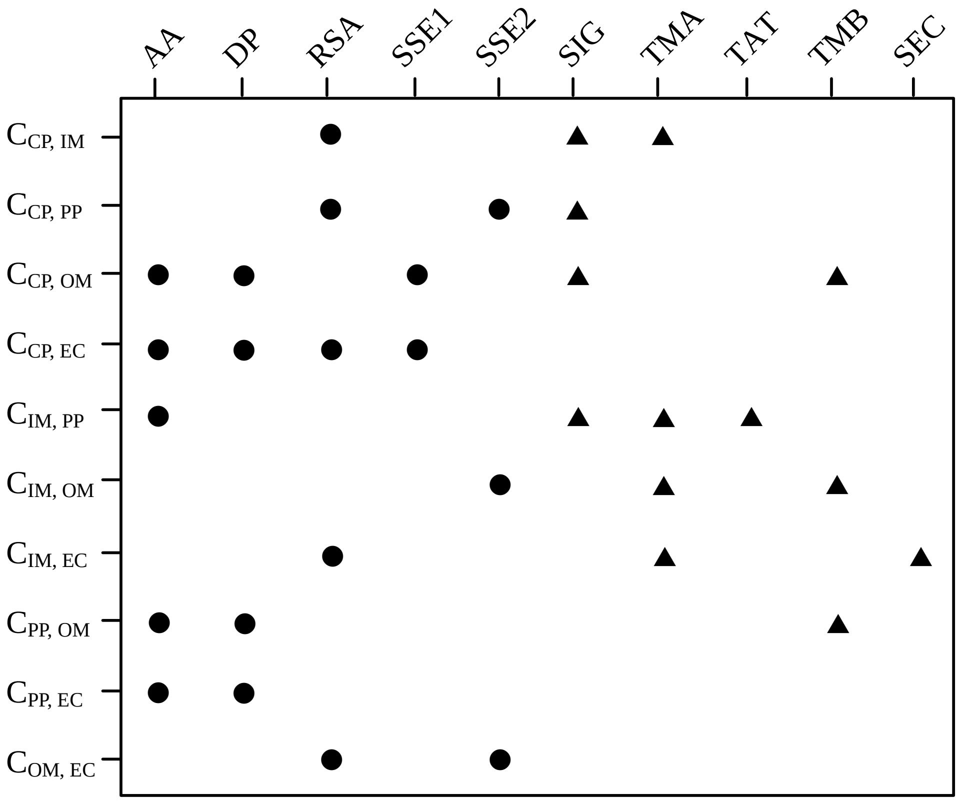

Figure 1S. Feature combinations derived from the PS1302 data set using a three-way data split procedure. Selected general and compartment-specific features are represented by filled circles and triangles, respectively.

3. THE SECOND ENCODING SCHEME FOR SECONDARY STRUCTURE ELEMENTS

To depict secondary structure elements (SSE), i.e., α-helix (H), β-strand (E), and loop (L), in a protein, three descriptors, composition (C), transition (T), and distribution (D), are used to encode predictions from HYPROSP II using Equations (1), (2), and (3), respectively [1].

$$C_i = (n_i / N) \times 100\%,\ \sum_i C_i = 100\%,\ i \in \{\mathrm{H, E, L}\} \tag{1}$$

$$T_{i \leftrightarrow j} = \left[t_{i \leftrightarrow j} / (N-1) \right] \times 100\%,\ i, j \in \{\mathrm{H, E, L}\},\ i \neq j \tag{2}$$

$$D_i^{p\%} = \left(d_i^{p\%} / N \right) \times 100\%,\ i \in \{\mathrm{H, E, L}\},\ p = \{1, 25, 50, 75, 100\}, \tag{3}$$

where C_i represents the composition of SSE type i; n_i is the number of SSE type i in a protein; N is the total number of amino acid residues in a protein; $T_{i \leftrightarrow j}$ measures the transition between SSE type i and j; $t_{i \leftrightarrow j}$ is the number of transitions between SSE type i and j; $D_i^{p\%}$ gives the distribution of $p\%$ located SSE type i; and $d_i^{p\%}$ is the position of $p\%$ located SSE type i in a protein.

For illustration purposes, a hypothetical SSE sequence is shown in Figure 2S. The sequence includes 12 α-helix residues (n_H = 12) and 8 β-strand residues (n_E = 8). The percent compositions are calculated as follows: $n_H / (n_H + n_E + n_L) \times 100\% = 60.0\%$ for H, $n_E / (n_H + n_E + n_L) \times 100\% = 40.0\%$ for E, and $n_L / (n_H + n_E + n_L) \times 100\% = 0.0\%$ for L. These three numbers represent the first descriptor, C. The second descriptor, T, characterizes the percent frequency that amino acids of a particular secondary structure element type are followed by a different type. In this case, there are 4 transitions of H to E or E to H, $T_{H \leftrightarrow E}$ is represented by (4 / 19) × 100.0% =

21.1%. $T_{H\leftrightarrow L}$ and $T_{E\leftrightarrow L}$ are equal to 0.0%, respectively. The third descriptor, D, measures the chain length within which the first, 25%, 50%, 75%, and 100% of the amino acids of a particular secondary structure element type are located. In Figure 1S, the first percent residue of α-helices coincides with the beginning of the chain, so the $D_H^{1\%}$ descriptor equals 0.0%. Twenty-five percent of α-helix residues are contained within the first 3 residues of the protein chain, so the second number equals (3 / 20) $\times$ 100.0% = 15.0%. Fifty percent of α-helix residues are within the first 11 residues of the chain; thus, the third number is (11 / 20) $\times$ 100.0% = 55.0%. The fourth and fifth numbers of the distribution descriptor are 70.0% and 100.0%, respectively. Similarly, analogous numbers for β-strand and loop residues are calculated.

SSE sequence	**H**	**H**	**H**	**H**	**E**	**E**	**E**	**E**	**E**	**H**	**H**	**H**	**H**	**H**	**E**	**E**	**E**	**H**	**H**	**H**
SSE index	1				5					10					15					20
Index for **H**	1	2	3	4						5	6	7	8	9				10	11	12
Index for **E**					1	2	3	4	5						6	7	8			
**H** ↔ **E** transitions					│					│					│			│		

Figure 2S. A hypothetical SSE sequence that illustrates the derivation of the SSE2 feature vector for a protein.

REFERENCE

1. Dubchak I, Muchnik I, Holbrook SR, Kim SH: **Prediction of protein folding class using global description of amino acid sequence**. *Proc Natl Acad Sci USA* 1995, **92**(19):8700-8704.
